# Development and Implementation of a Dynamically Updated Big Data Intelligence Platform Using Electronic Medical Records for Secondary Hypertension

**DOI:** 10.31083/j.rcm2503104

**Published:** 2024-03-12

**Authors:** Nanfang Li, Qing Zhu, Yujie Dang, Yin Zhou, Xintian Cai, Mulalibieke Heizhati, Delian Zhang, Xiaoguang Yao, Qin Luo, Junli Hu, Guoliang Wang, Yingchun Wang, Menghui Wang, Jing Hong

**Affiliations:** ^1^Hypertension Center of People's Hospital of Xinjiang Uygur Autonomous Region; Xinjiang Hypertension Institute; National Health Committee Key Laboratory of Hypertension Clinical Research; Key Laboratory of Xinjiang Uygur Autonomous Region “Hypertension Research Laboratory”; Xinjiang Clinical Medical Research Center for Hypertension (Cardio-Cerebrovascular) Diseases, 830001 Urumqi, Xinjiang, China; ^2^Medical Department, Yidu Cloud (Beijing) Technology Co., Ltd., 100191 Beijing, China

**Keywords:** secondary hypertension, database, electronic medical records

## Abstract

**Background:**

The accurate identification and diagnosis of secondary hypertension is critical,especially while cardiovascular heart disease continues to be the leading cause of death. To develop a big data intelligence platform for secondary hypertension using electronic medical records to contribute to future basic and clinical research.

**Methods:**

Using hospital data, the platform, named Hypertension DATAbase at Urumchi (UHDATA), included patients diagnosed with hypertension at the People’s Hospital of Xinjiang Uygur Autonomous Region since December 2004. The electronic data acquisition system, the database synchronization technology, and data warehouse technology (extract–transform–load, ETL) for the scientific research big data platform were used to synchronize and extract the data from each business system in the hospital. Standard data elements were established for the platform, including demographic and medical information. To facilitate the research, the database was also linked to the sample database system, which includes blood samples, urine specimens, and tissue specimens.

**Results:**

From December 17, 2004, to August 31, 2022, a total of 295,297 hypertensive patients were added to the platform, with 53.76% being males, with a mean age of 59 years, and 14% with secondary hypertension. However, 75,802 patients visited the Hypertension Center at our hospital, with 43% (32,595 patients) being successfully diagnosed with secondary hypertension. The database contains 1458 elements, with an average fill rate of 90%. The database can continuously include the data for new hypertensive patients and add new data for existing hypertensive patients, including post-discharge follow-up information, and the database updates every 2 weeks. Presently, some studies that are based on the platform have been published.

**Conclusions:**

Using computer information technology, we developed and implemented a big database of dynamically updating electronic medical records for patients with hypertension, which is helpful in promoting future research on secondary hypertension.

## 1. Introduction

Hypertension is a pressing public health concern worldwide and a major modifiable risk factor for cardiocerebrovascular disease and renal failure [1]. Hypertension can be divided into primary hypertension with unknown causes and secondary hypertension with clear causes [2]. Previously, primary hypertension was believed to be the most common form of the disease, with secondary hypertension only accounting for 5–10% of cases [3, 4, 5, 6]. However, with a comprehensive understanding of the etiology of hypertension and the improvements in clinical diagnostic techniques, the proportion of secondary hypertension has been found to significantly exceed current expectations [4, 7, 8]. According to existing research and clinical observations, secondary hypertension often leads to more serious target organ damage than primary hypertension [9, 10, 11, 12, 13]. Therefore, early detection and treatment of secondary hypertension are of crucial clinical significance.

However, the etiology of secondary hypertension is complex, involving cardiovascular diseases, endocrine diseases, kidney diseases, sleep, mental illness, and other disciplines (**Supplementary Table 1**). Thus, it is necessary to pay attention to the standardized procedures during screening to avoid the occurrence of missed diagnoses and misdiagnoses. It is not advisable to blindly screen everyone for secondary hypertension since it will result in a waste of medical resources and cause an economic burden on both the patients and society. Therefore, it requires the involvement of specialized departments, teams, and clinicians [2, 14].

**Fig. 1.**
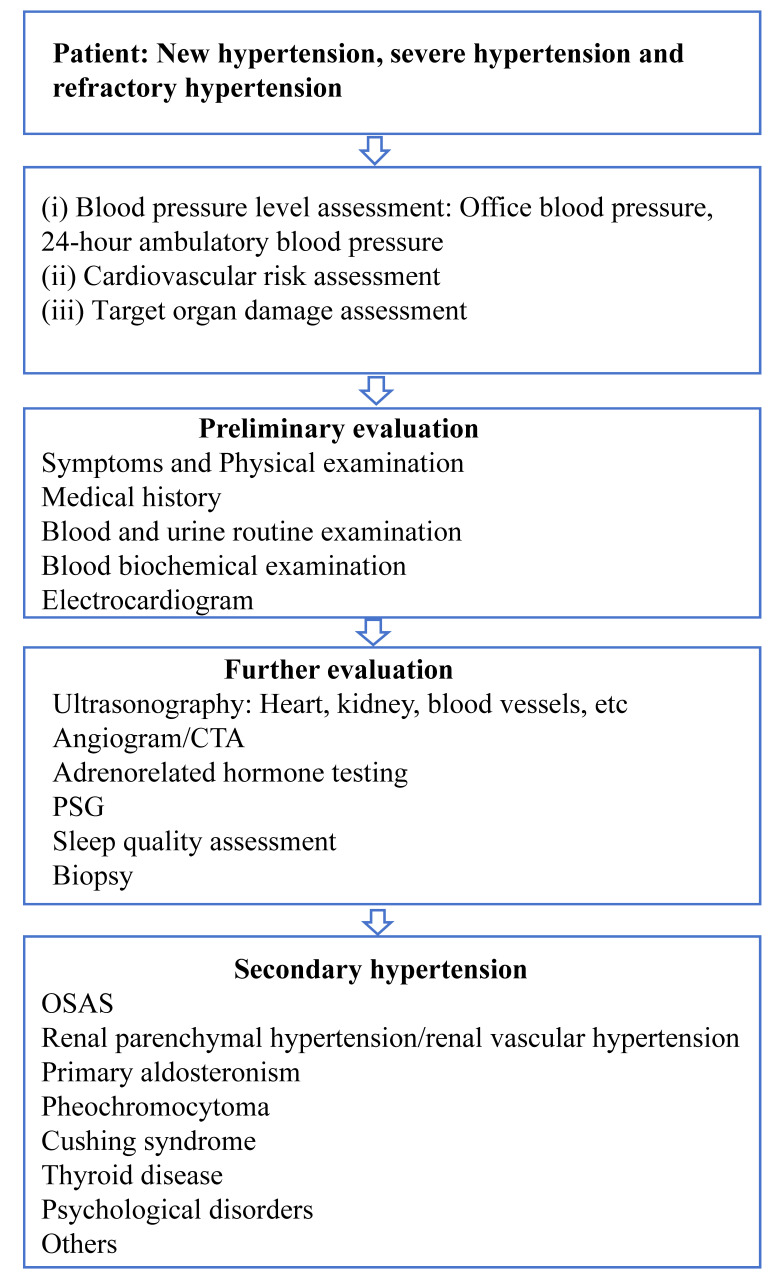
**Flow chart for diagnosis and treatment of hypertensive patients**. CTA, computed tomography angiography; PSG, polysomonography; OSAS, obstructive sleep apnea syndrome.

Since 1997, the Hypertension Center at the People’s Hospital of Xinjiang Uygur Autonomous has gradually established a platform for secondary hypertension screening, diagnosis, and treatment (**Supplementary Figs. 1,2**). A detailed description of our center is provided in the **Supplementary Materials**. Since its establishment, the Hypertension Center has provided diagnostic and treatment services for each patient with hypertension from various regions of Xinjiang. Among all patients who visit our center, some do so to clarify their hypertension etiology, while others are refractory hypertension patients who have been referred by primary hospitals. For patients who visit our hypertension center, we first judged whether they were clearly hypertensive and measured their blood pressure level according to their home blood pressure, office blood pressure, or ambulatory blood pressure results. Next, patients were given corresponding examinations to evaluate their cardiovascular risk factors and the damage to their target organs (Fig. 1). In addition, doctors with 5 years or more working experience judge whether there are screening clues for secondary hypertension according to any symptoms and signs demonstrated by the patients and the results of routine examination (**Supplementary Table 2**). Patients with positive clues will be screened for secondary hypertension (Fig. 1). All our test items were performed in accordance with the standards of international guidelines [15], such as polysomonography (PSG) test preparation and interpretation standards for obstructive sleep apnea syndrome (OSAS) [2, 7, 8], and the requirements of renin aldosterone determination on drugs and disease status during primary aldosteronism (PA) screening [2, 8, 16, 17, 18]. The procedures of screening, diagnosis, and treatment for common secondary hypertension are shown in **Supplementary Figs. 3,4,5,6**.

At present, it is one of the main problems that we need to solve to determine the main influencing factors of secondary hypertension on target organs and cardiovascular diseases. In addition, there are still many unknown problems to be solved in the different types of secondary hypertension, such as the different degrees of target organ damage, and different treatment methods. It is also urgent to optimize the process of PA diagnosis and lateral typing. Therefore, we analyzed the data for every secondary hypertension case to solve these various clinical problems. However, in previous clinical research, medical data were dispersed across several business information systems within medical institutions, which resulted in data structure diversity, unstable quality, and inconsistent data standards. Detailed data collation is highly dependent on the manual operation of doctors. However, the cumbersome manual entry process leads to high error rates, difficulties in organizing the data, and the arrangement of complex clinical events, such as long research feasibility assessments. Consequently, this array of issues can hinder the effective integration and utilization of medical data [19]. In 2019, a project was launched in our hospital to develop a big intelligent database for hypertension by integrating current electronic medical records to provide high-quality basic and clinical research on hypertension, especially secondary hypertension.

## 2. Methods

### 2.1 Data Source and Procedure of Hypertension-Specific Database 

Data were obtained from electronic medical records for hypertensive patients at the People’s Hospital of the Xinjiang Uygur Autonomous Region in China.

**Fig. 2.**
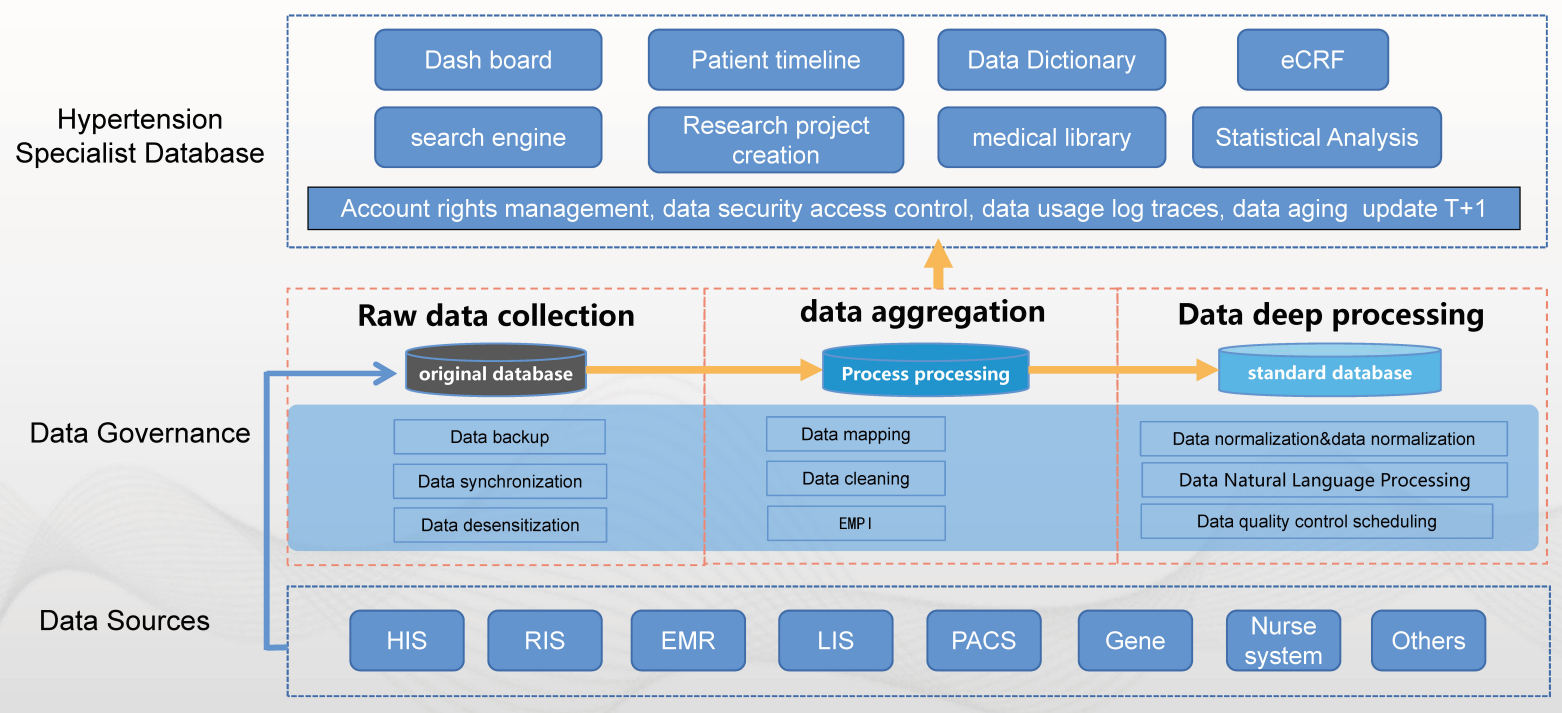
**Framework of database construction**. HIS, hospital information system; EMR, electronic medical record; RIS, radiology information system; LIS, laboratory information system; PACS, picture archiving and communication system; eCRF, electronic case report form; EMPI, Enterprise Master Patient Index.

The project included four procedures: (a) full-scale, multi-source, and heterogeneous data extraction and integration in hospitals; (b) data structurization and normalization; (c) development of hypertension-specific standard data elements; (d) construction of functional modules on the platform. In terms of integrating multi-source and heterogeneous data, the database synchronization technology of the scientific research big data platform and data warehouse technology (extract–transform–load, ETL) is used to synchronize and extract the data from various business systems in the hospital, to realize the collection, aggregation, and cleaning of multi-source and heterogeneous data for multiple information systems in the hospital (Fig. 2) [20, 21, 22]. Concurrently, it supports the import of information islands and data from external sources to ensure that eligible cases are automatically and continuously entered into the database and a full, continuous, complete, and reusable data asset is formed. In the process of data integration, it is worth noting that the corresponding relationship between the hypertension-specific disease data set, business activity, and the data source needs to be systematically elucidated. Conversely, it ensures that the hypertension-specific disease scientific research database comprehensively covers all information systems in the hospital and ensures comprehensive and effective data collection. Additionally, the traceability of individual data (business systems, business activities) can be ensured (**Supplementary Fig. 7**). Cardiologists, professional doctors with deputy senior titles or above from our hypertension center, informatics technicians, and software engineers from a technology company (Yidu Cloud Company) were all involved in this project. This database construction scheme was approved by the Ethics Committee for our hospital in 2019.

### 2.2 Establishment of Hypertension-Specific Standard Data Elements 

To define all the variables in the platform, standard data elements were established, including 26 data element modules with a total of 1458 common and hypertension-specific variables (**Supplementary Fig. 8** and **Supplementary Table 3**). Hypertension-specific data elements were constructed with reference to international standards and local databases and terminology standards, as shown in **Supplementary Table 4**.

### 2.3 Data Extraction and Integration

Patients with hypertension who had visited our hospital since 2004 (our hospital began to use electronic medical records in 2004) were included in the platform, including patients with primary hypertension and secondary hypertension. Inclusion criteria: Visit type = hospitalization, and the diagnosis name during the visit contains “hypertension/HT” or an international classification of diseases (ICD)10 code containing “I10”–“I15”. For each patient, data were extracted from 13 EHR systems [20] and a follow-up system (**Supplementary Figs. 2,7**). Additionally, it was also connected to the physical examination information and biological sample bank system.

Data extractions were performed using ETL according to the predefined standard data elements [20]. In this process, we used the unique identification number provided by the Enterprise Patient Master Index (EPMI) to consolidate each record for the same patient from 16 systems into one record.

### 2.4 Data Structurization and Normalization

We used natural language processing (NLP) and multiple machine learning models for data structurization and normalization. Detailed processes of NLP are presented in previous research [20]. At present, a total of 1458 variables have been produced: 920 first-level variables, 440 second-level variables, and 98 third-level variables. By constantly modifying and constantly optimizing, the precision and recall rate for most variables can reach more than 95%.

### 2.5 Functional Modules on the Platform

To facilitate the research, eight functional modules were designed for the platform, including case retrieval and extraction, project creation, statistical analysis, follow-up, etc.

### 2.6 Patient Privacy and Platform Security

To protect the privacy and data security of each patient, we desensitized their personal privacy information and established different permission sets specific to each patient’s need [19, 23].

## 3. Results

### 3.1 Overview of the Platform

**Fig. 3.**
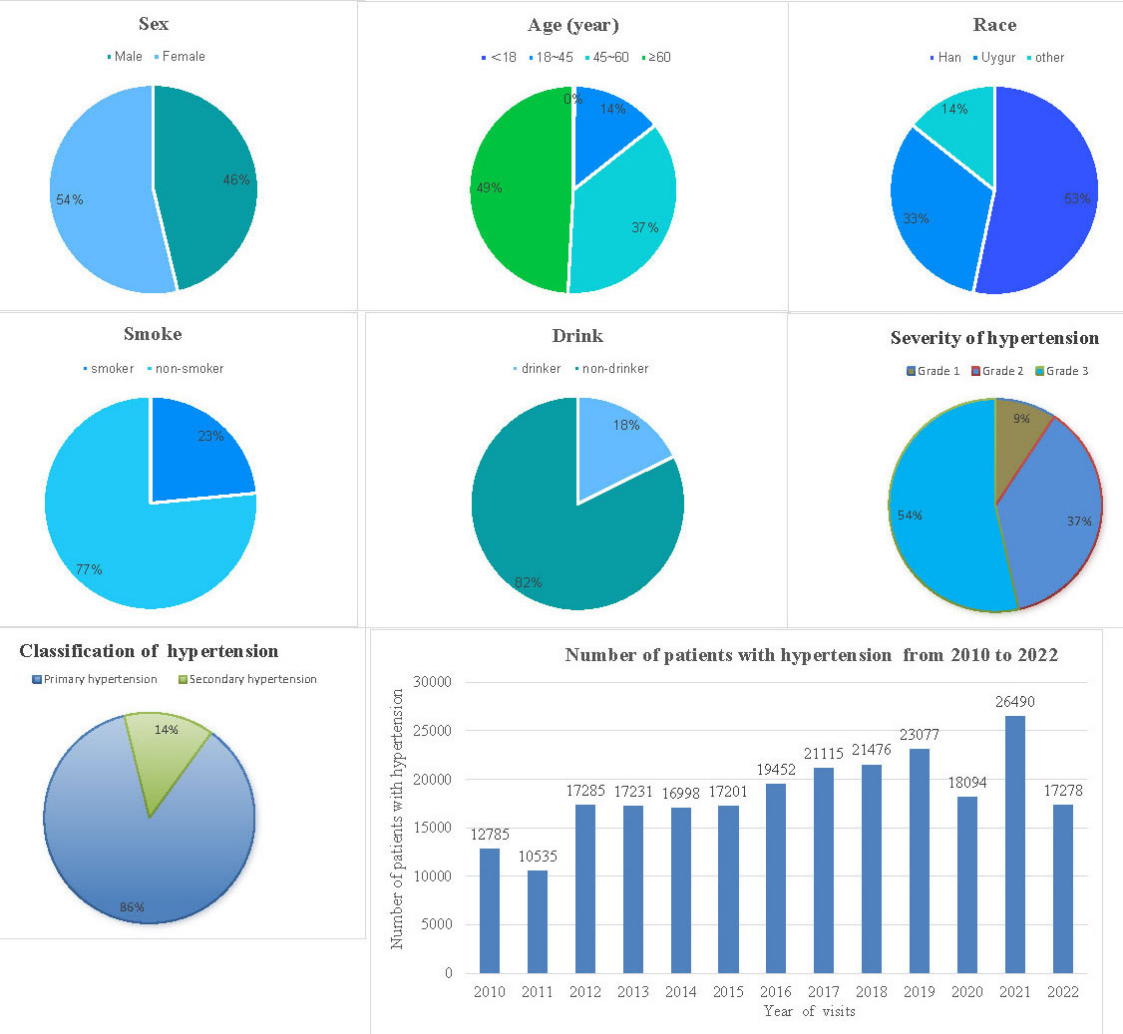
**The basic characteristics of hypertensive patients on the platform**.

From December 17, 2004, to August 31, 2022, a total of 295,297 hypertensive patients were added to the platform, with 53.76% being males, with a mean age of 59 years, and 14% with secondary hypertension (Fig. 3). However, 75,802 patients visited the Hypertension Center at our hospital, with 43% (32,595 patients) being successfully diagnosed with secondary hypertension. For the hypertension classification, 158,663 people (53.73%) were in grade 3, 109,998 people (37.25%) were in grade 2, and 26,665 people (9.03%) were in grade 1. From 2010 to 2022, the annual number of patients with hypertension gradually increased, and the number of patients affected during the epidemic decreased in 2020 (Fig. 3).

### 3.2 Examples of Advanced Search

To build a condition tree, advanced retrieval uses multi-dimensional inclusion and exclusion criteria to retrieve the diagnosis and treatment information for each patient. For example, if the age at treatment is ≥18, and the diagnosis name is hypertension, there are 254,672 eligible people. Then, a total number of hospitalizations of ≥ 2,108,109 people also meet the conditions (**Supplementary Fig. 9**).

### 3.3 eCRF

An electronic case report form (eCRF) can be created on the platform for prospective clinical trials and cohort studies. Moreover, through follow-ups and an extension of the hospital’s service function, the prognosis of the disease can be identified to a certain extent, thereby providing a more comprehensive understanding of the disease.

### 3.4 Platform Implementation Status

The platform was launched in December 2019 and has been adopted by several clinicians and graduate students. At present, more than 30 retrospective studies and 2 prospective cohort studies are either being performed or have been conducted based on the platform. Six studies have been published, involving studies on aldosterone and the brain and macrovascular complications [24, 25, 26, 27, 28, 29].

## 4. Discussion

A research disease database is an important tool for clinicians to collect and organize data and conduct scientific research. At present, the method of collecting and sorting data is still mainly manual, which provides some problems, such as heavy workload, low efficiency, high error rate, and difficulty sharing and utilizing collected and sorted data. Following the construction of the diversified scientific research disease database for clinical departments under the big data environment, a number of scientific research databases have also been produced based on electronic medical record system [30, 31, 32, 33, 34], which is the result of the continuous development and intersection of computer technology, information technology, and medicine. Moreover, CRF visual personalized customization and template management, data integration and integration, batch import, automation and auxiliary control filling, and multi-way data security management, have been implemented to assist the department in performing clinical research to the highest quality and efficiency.

Hypertension affects nearly 1.3 billion people worldwide, and the burden of cardiovascular and cerebrovascular diseases caused by hypertension remains first in the global disease burden list. In particular, damage to the target organs by secondary hypertension is more serious than in primary hypertension, meaning the mechanisms need to be explored and addressed through more clinical or basic research. Thus, our hospital has built a big data intelligence platform for hypertension research (hypertension-specialist database), named Hypertension DATAbase at Urumchi (UHDATA), to promote research on hypertension (including secondary hypertension), using the electronic data acquisition system, the database synchronization technology, and data warehouse technology (extract–transform–load, ETL) on the scientific research big data platform. Presently, data on nearly 300,000 hypertensive patients are available on the platform. To the best of our knowledge, this is currently the largest hypertension-specialized database and the largest database for secondary hypertension.

At present, most databases based on electronic medical record systems are specialized disease databases from a single center or hospital. Although it does not contain information on all patients of multiple centers or even an entire province, it contains more comprehensive and detailed data than found in electronic health insurance databases and commercial insurance databases, especially in real-world studies, which may need to control for many confounding factors to ensure the reliability of the study. In addition, the electronic medical record database contains more imaging data and laboratory indexes, which are very helpful for diagnosing and treating secondary hypertension and any associated complications [35, 36]. This will be very helpful in developing robots that can perform clinical diagnosis and treatment in the future.

Our big data platform has several limitations. First, the medical data archived within the database were not originally intended for secondary analysis. Thus, some missing values and inconsistencies may occur due to technical errors, system integration, and data preprocessing, which need continuous improvement. Second, there are currently no target standards for obtaining data elements or any definitions for those standard elements. Third, using such databases in either retrospective studies or real-world research would not replace the need to perform randomized controlled trials. However, they could serve as an important tool to supplement the contributions of trials for evidence-based medicine. Finally, it currently only exists as a local database from a single institution. Thus, to address the problem of data sharing, the data from multiple hospitals should be shared and implemented in the future.

## 5. Conclusions

We developed and implemented a big database of electronic medical records for hypertension that dynamically updates, and offers an important perspective on the future study of secondary hypertension.

## Data Availability

The datasets for this study are available from the corresponding author on reasonable request.

## Supplementary Material

Supplementary material associated with this article can be found, in the online version, at https://doi.org/10.31083/j.rcm2503104.
