# Supplementary material for: Development and Implementation of a Dynamically Updated Big Data Intelligence Platform Using Electronic Medical Records for Secondary Hypertension
Source: Rev Cardiovasc Med. 2024 Mar 12;25(3):104. doi: 10.31083/j.rcm2503104 (PMC11263842; doi:10.31083/j.rcm2503104)
Supplement: Supplementary file 1 [file 2153-8174-25-3-104-s1.pdf]

Supplementary material

Development and implementation of a dynamically updated big data intelligence platform from electronic medical records for secondary hypertension

Platform for secondary hypertension screening, diagnosis and treatment

Since 1997, Hypertension Center of the People's Hospital of Xinjiang Uygur Autonomous has gradually established a platform for secondary hypertension screening, diagnosis and treatment(**Figure S1**). Currently, Hypertension Center has 5 outpatient clinics, including General Outpatient, Specialized Service and Expert Outpatient Service, and has 180 beds distributed in 4 wards. On the basis of the large-scale comprehensive medical examination platform, the Hypertension Center has set up the hypertension-oriented physical examination unit(including Dynamic Blood Pressure Chamber, Fundus Photography Room, the Arterial Elastic Testing Room, OSA Monitoring Room and Vascular Intervention Room) and the department of biochemical inspection （including endocrine hormone test, vasoactive substances test, cardiovascular risk factor evaluation and genetic test（**Figure S2**）.This Center has initiated research on sleep disorders and hypertension since 1999, and the Sleep Monitoring Room thereof has been officially established since 2004. Currently, there are 5 sets of polysomnography (PSGs) with 52 channels, 20 portable sleep monitors with 26 channels and 10 initial screening machines for sleep monitoring. Up to now, more than 30,000 patients have been undergone PSG test, over 8,000 patients have undergone noninvasive Continuous Positive Airway Pressure (non-CPAP) treatment and more than 3,000 patients have undergone oral appliance treatment. The Vascular Intervention Center is equipped with a fully functional Syngo X workstation Siemens large full-size C arm, we have performed more than 4000 cases of hypertension related interventional diagnosis and treatment, including adrenal venous blood sampling

(AVS), inferior petrosal sinus sampling (IPSS), arteriocerebral angiography renal arteriography and stenting, adrenal artery alcohol ablation and left renal vein stenting.

Our hypertension Center enjoys a team of excellent experts on hypertension, which consists of 82 medical staff (30 doctors and 52 nurses), among which 16 are associate chief physicians or above, and 93.54% of the doctors have a master's degree or above.They have extensive experience in the diagnosis and study of secondary hypertension.

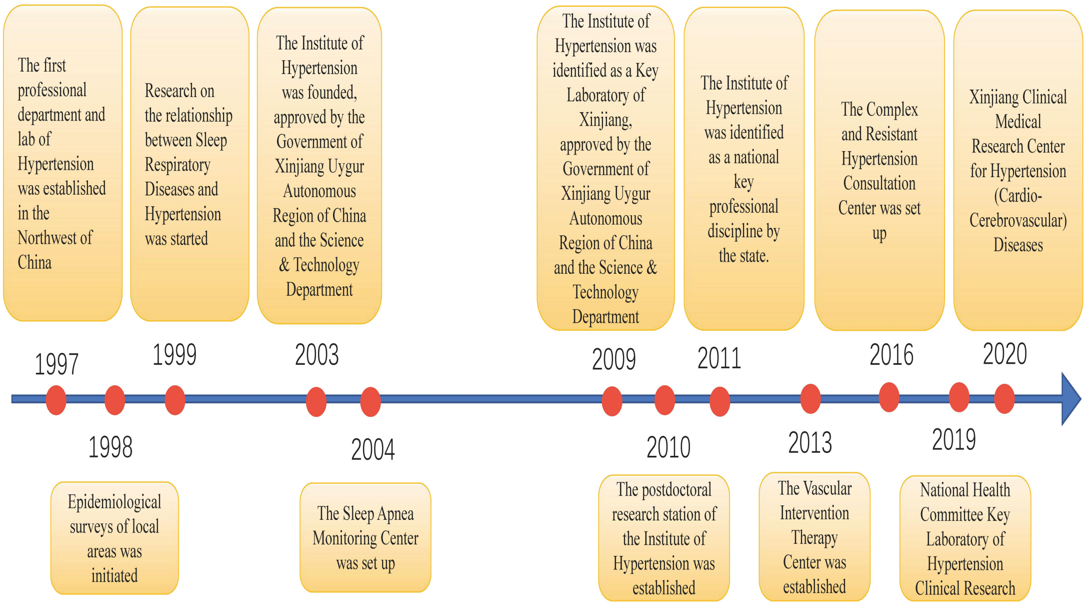

Figure S1: Development of hypertension center (created a platform for screening, diagnosis and treatment of secondary hypertension).

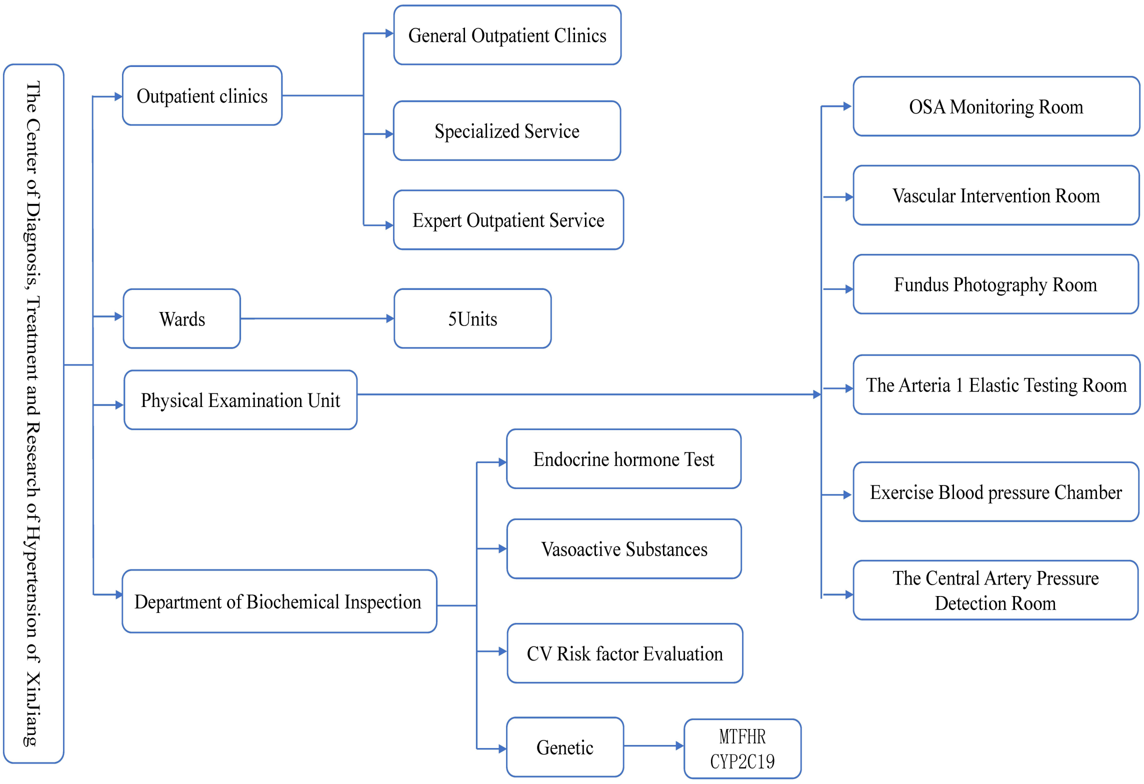

Figure S2: Framework of Hypertension Center and Platform for screening, diagnosis and treatment of secondary hypertension.

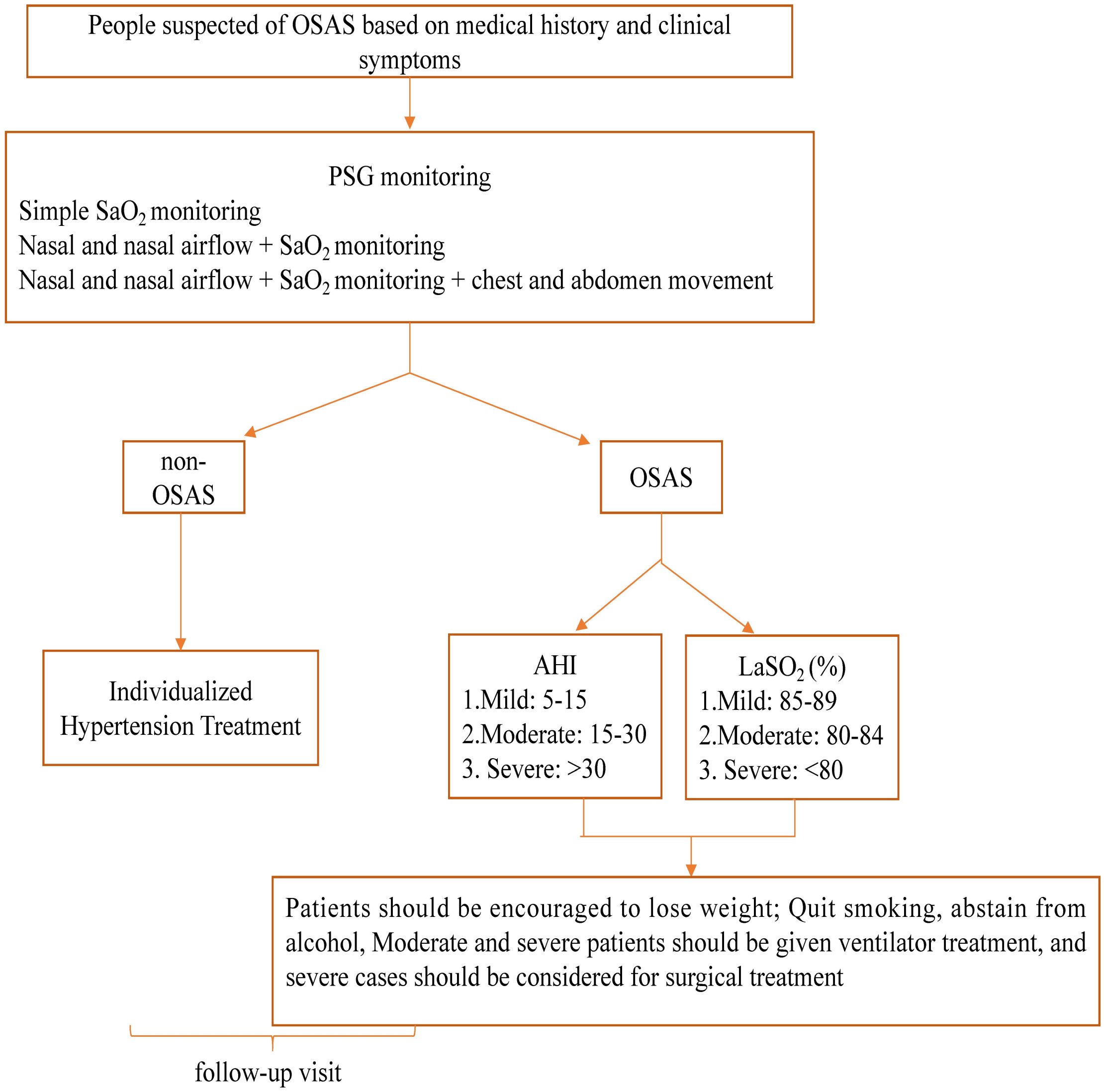

Figure S3 Flow chart of screening, diagnosis, treatment, and follow-up for obstructive sleep apnea syndrome（OSAS）

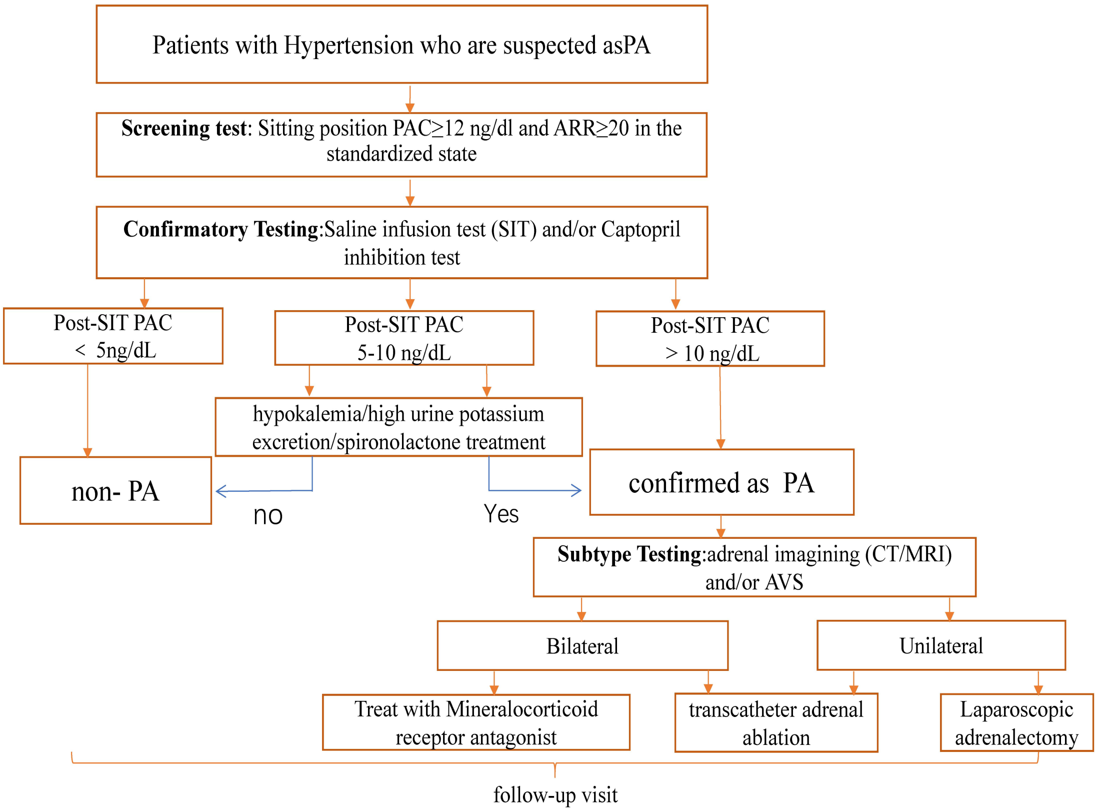

Figure S4 Flow chart of screening, diagnosis, treatment, and follow-up for primary aldosteronism (PA)

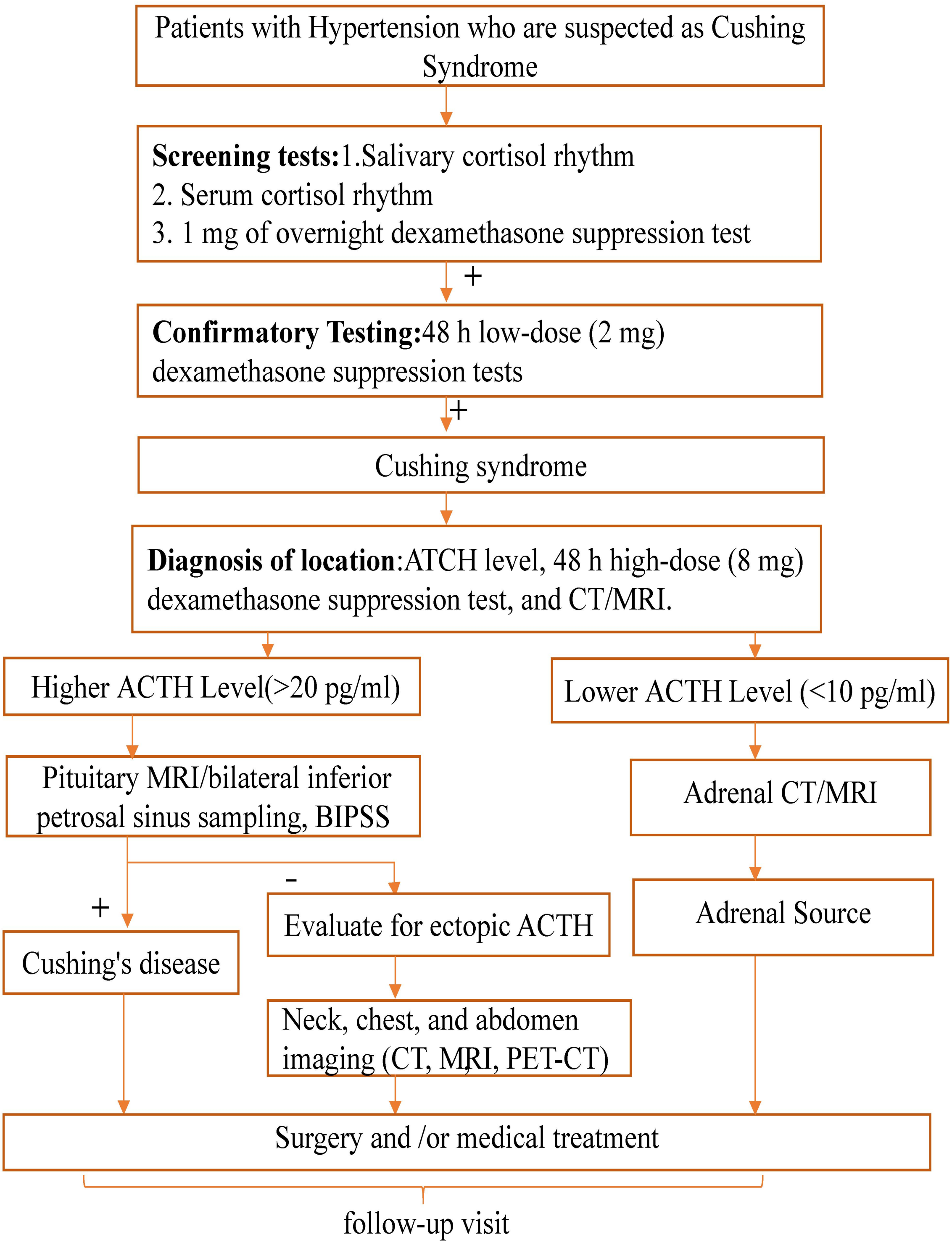

Figure S5 Flow chart of screening, diagnosis, treatment, and follow-up for Cushing Syndrome

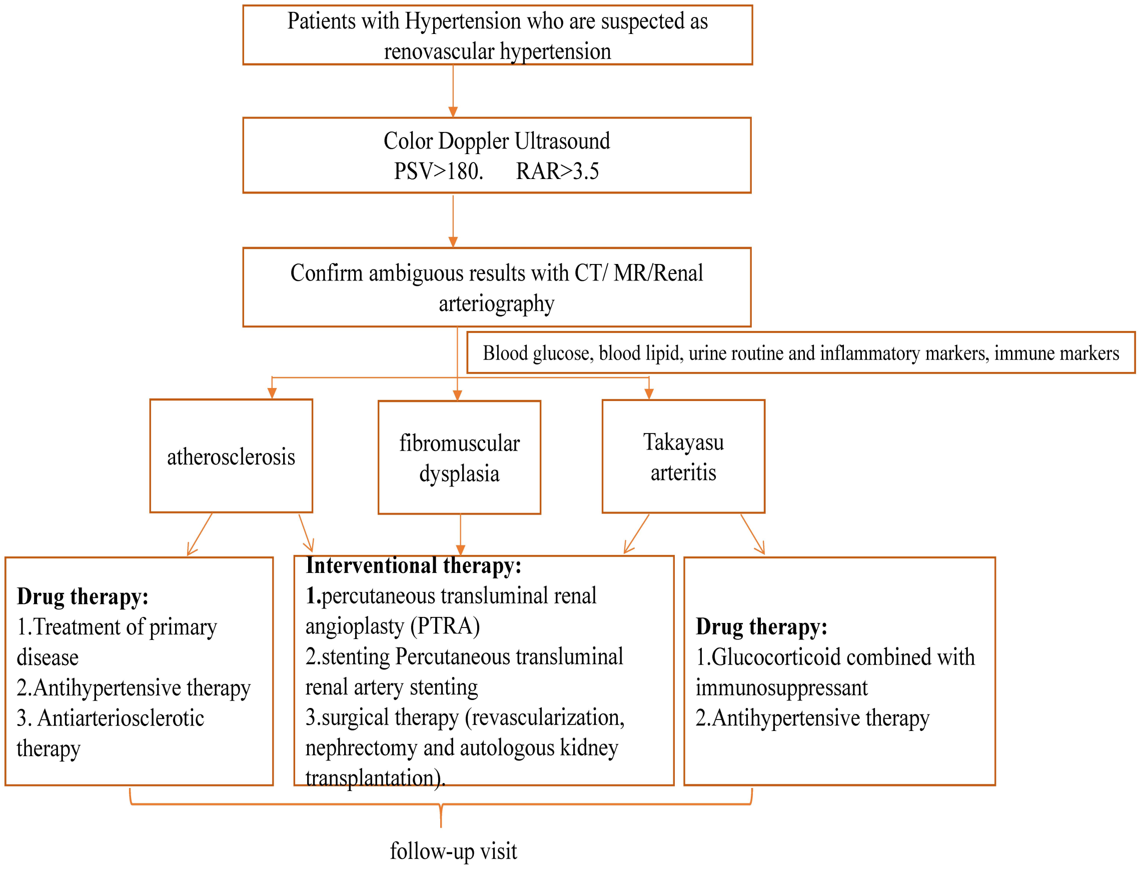

Figure S6 Flow chart of screening, diagnosis, treatment, and follow-up for Renovascular hypertension

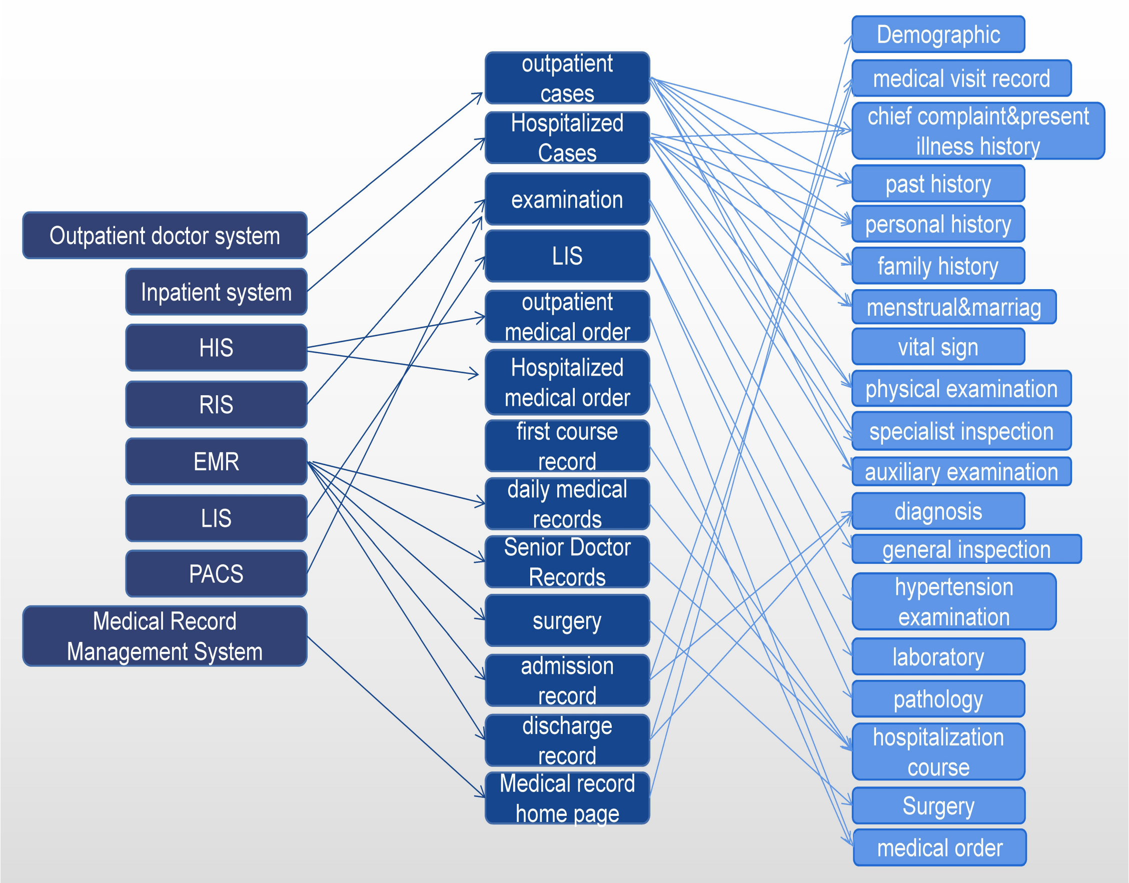

Figure S7 The development process of data platform

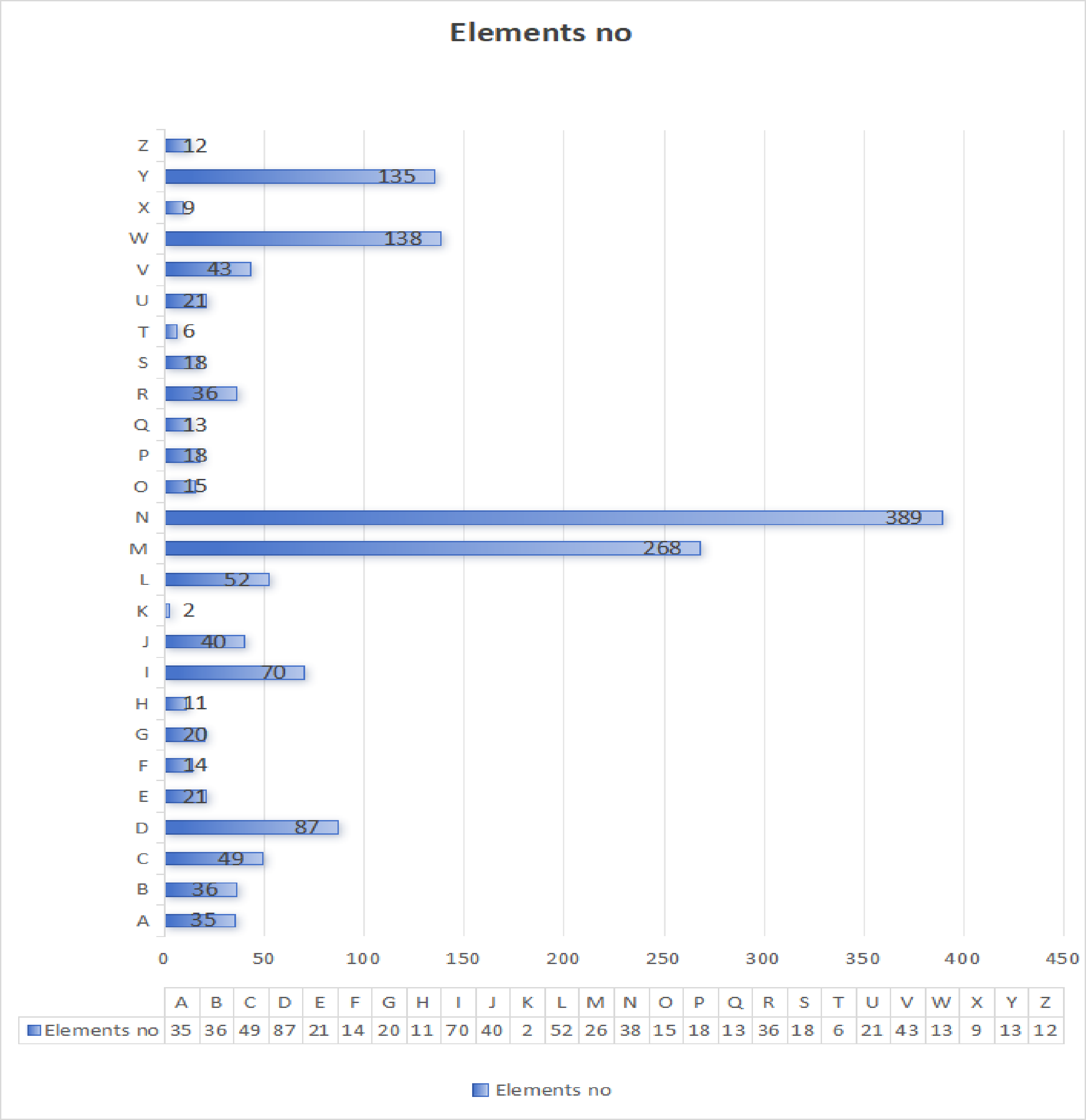

	A	B	C	D	E	F	G	H	I	J	K	L	M	N	O	P	Q	R	S	T	U	V	W	X	Y	Z
Elements no	35	36	49	87	21	14	20	11	70	40	2	52	26	38	15	18	13	36	18	6	21	43	13	9	13	12

Figure S8: Modules and standard data elements

A,Demographic; B,Medical visit record; C,Chief complaint&present illness history; D,Past history; E,Personal history; F,Family history; G,Menstrual&marriage; H,Vital sign; I,Physical examination; J,Specialist inspection; K,Auxiliary examination; L,Diagnosis; M,General inspection; N,Hypertension examination; O,Laboratory; P, Pathology; Q,Molecular immune markers; R,Hospitalization course; S,Surgery; T,Discharge record; U,Follow-up ;V,Medical order; W,Inpatient evaluation; X,AE; Y,Nursing records; Z,Treatment overview

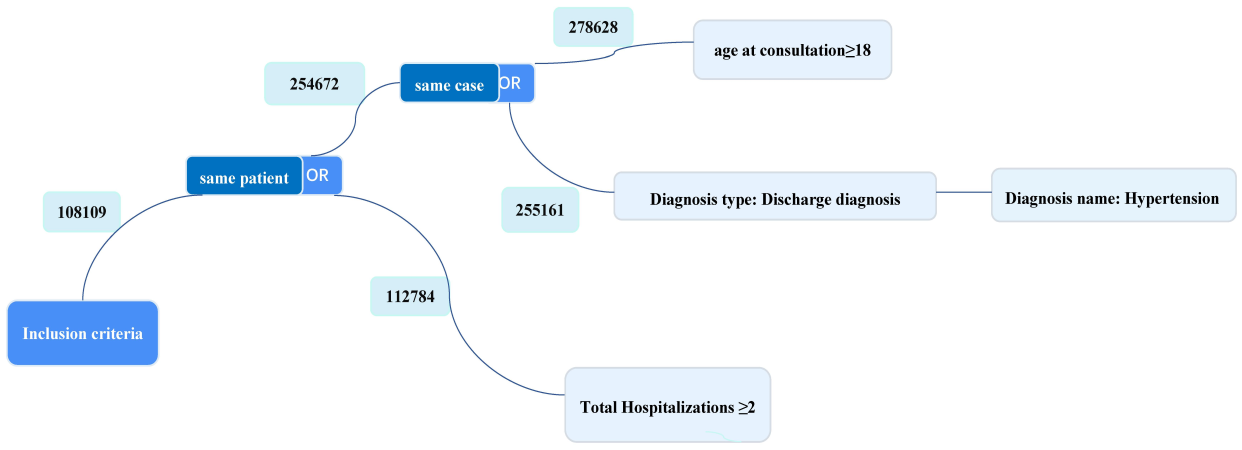

Figure S9 Advanced search procedure

Table S1: Common causes of secondary hypertension

Causes		
**Obstructive sleep apnea（OSA）**		
**Endocrine**	Pituitary disease	Various types of pituitary tumors
	Thyroid disease	Hyperthyroidism
		Hypothyroidism
	Parathyroid disease	Hyperparathyroidism
	Adrenal disease	Primary aldosteronism (PA)
		Cushing syndrome (CS)
		Pheochromocytoma/paragangliomas (PPGL)
	Congenital adrenal hyperplasia	11beta-hydroxylase and
		17alfa-hidroxylase deficiency
	Pancreatic disease	Acute Pancreatitis
		Insulinoma
		Chronic Pancreatitis
	Ovarian disease	polycystic ovarian syndrome
	Other	Type 1 Multiple Endocrine Neoplasia (MEN1)
		Deoxycorticosterone-producing tumor
**Renal**	Renal Parenchymal hypertension	Acute Glomerulonephritis
		Obesity-Related Glomerulopathy
		Lupus Nephritis
		Hydronephrosis, Obstructive Nephropathy
		Pyelonephritis
		Reflux Nephropathy
		Kidney Tumor
		IgA nephropathy
		Membranoproliferative glomerulonephritis
		Focal segmental glomerulosclerosis
		Chronic interstitial nephritis
		Dialytic
		Kidney Transplantation
	Renovascular hypertension	Atherosclerosis
		Fibromuscular dysplasia (FMD)
		Takayasu arteritis
		Polyarteritis nodosa
**Autoimmune disease**		Systemic lupus erythematosus
		Systemic sclerosis
		Sjogren syndrome
		Various types of vasculitis
**Drug-induced**		glucocorticoid
		contraceptive
		Immunosuppressive agent: cyclosporin
		Liquorice tablets

**Others**	Antidepressive drugs<br>Ephedrine Hydrochloride<br>anxiety<br>Liddle’s syndrome<br>**Gordon’ syndrome**<br>Coarctation of the aorta<br>Aorta coarctation<br>Hypertensive disorders of pregnancy<br>......

Table S2: Screening clues for common secondary hypertension

Secondary hypertension	History and symptom characteristics	Signs
Sleep apnea hypopnea syndrome	Snoring during sleep, frequent episodes of apnea during sleep, dreaminess, enuresis, night angina; excessive daytime sleepiness, fatigue, memory loss, decreased ability to work, etc	Obesity, short neck, small jaw deformity, dark skin, cyanosis, fat tongue, etc.
Pituitary adenoma	Gigantism, acromegaly, galactorrhea; headache, nausea, vomiting, double vision, etc.	Abnormal physical appearance; visual field defect, etc.
Thyroid disease	1. Hyperactivity: Good appetite, palpitations, hyperhidrosis, irritability, etc.<br>2. Hypoglycemia: dropsy, depression, etc.	1. Convex eyes and flapping pterodactyl tremor of both upper limbs<br>2. Mucous edema, etc.
Hyperparathyroidism	Recurrent urinary stone, bone pain, etc.	No special
Primary aldosteronism	Muscle weakness, palpitations, abdominal distension, etc.	No special
Hypercortisolism	Mood disorders, menstrual irregularities, muscle weakness, etc.	Weight gain, abdominal striae, hirsutism, dorsal and supraclavicular fat, fragile skin, etc.
Pheochromocytoma	Paroxysmal hypertension, palpitations, excessive sweating, severe headache, etc	Emaciation, rapid heart rate, postural hypotension, moist skin, etc.
Renal parenchymal hypertension	Fever, edema, hematuria, foam urine, renal area pain, etc.	Sunken edema of the face or limbs; percussion pain in renal region; bladder percussion area enlargement, etc.
Renal vascular hypertension	Diastolic pressure significantly increased, prone to pulmonary edema, etc.	There are vascular murmurs on both sides of the umbilicus of the upper abdomen or the lumbar ridges
Aortic disease	Double lower limb weakness, difficulty in breathing, etc.	Aortic murmur; abnormal blood pressure distribution in the extremities: double upper limb blood pressure, double lower limb blood pressure, etc.

Table S3 Modules and standard data elements

Serial number	Baseline name	Baseline field content
1	Patient demographic information	Name, gender, ethnicity, nationality, date of birth, occupation category, telephone number, ABO blood type, hospital number, outpatient number, etc.
2	medical records	Type of visit, date of visit or admission, department of visit or admission, age of visit, main diagnosis, name of main diagnosis ICD10, pathological diagnosis, etc.
3	History of present illness	Admission time, history of present illness, normal blood pressure level, highest blood pressure, course of hypertension, name of previous medication, etc.
4	Past history	Admission time, name of previous disease, hypertension, duration of hypertension, diabetes, history of infectious diseases, history of cerebral hemorrhage, etc.
5	personal history	Admission time, smoking, smoking age (years), daily smoking volume (cigarettes/day), quitting smoking, duration of quitting smoking, drinking alcohol, drinking age (years), etc.
6	family history	Admission time, family history, name of family disease, kinship of family disease, family history of malignant tumor
7	History of menstruation, marriage and childbearing	Time of admission, family history, name of family disease, kinship of family disease, family history of malignant tumor
8	vital signs	Check time, body temperature, heart rate, respiratory rate, diastolic blood pressure, systolic blood pressure, pulse, height, weight, SpO2, body mass index (BMI)
9	physical examination	Admission time, admission respiratory rate, admission diastolic blood pressure, admission systolic blood pressure, admission pulse, double lung percussion sound, whether there is dry rales in the lungs, whether there is arrhythmia, etc.
10	Specialist inspection	Heart rate, third heart sound, galloping rhythm, period of heart murmur, location of heart murmur, nature of heart murmur, classification of heart murmur, etc.
11	Auxiliary examination	Admission time, auxiliary examination
12	diagnosis	Time of visit, date of diagnosis, type of diagnosis, name of diagnosis, name of diagnosis ICD10, source of diagnosis, whether secondary hypertension, hypertension classification, hazard stratification, etc.

13	Examination	Echocardiography, 24-hour Holter, ECG, Abdominal Ultrasound, Thyroid Ultrasound, Coronary CTA, Renal Artery CTA, Renal Artery Ultrasound, etc.
14	Test	Blood routine, urinalysis, stool routine, biochemical test, coagulation test, blood gas analysis, sex hormone test, thyroid function test, autoantibody test, etc.
15	pathology	Examination date, pathological application number, pathological examination number, examination name, material findings, pathological findings, pathological conclusion, collection site, immunohistochemical results
16	Course record	First disease course record, first postoperative course record, daily disease course and superior ward round records, other disease course records, intravascular pressure measurement information
17	Operation	Operation start time, operation end time, operation name (original value), preoperative diagnosis (original value), postoperative diagnosis (original value), surgical procedure description, etc.
18	Discharge records	Date of admission, date of discharge, condition of admission, description of diagnosis and treatment process, condition of discharge, doctor's order for discharge
19	Follow up	Date of follow-up, method of follow-up, status of follow-up
20	Doctor's order	Non-drug doctor's order, drug doctor's order, Chinese herbal medicine doctor's order
21	Inpatient evaluation	90-item symptom list (scl90 test report), Hamilton Anxiety Scale
22	Nursing records	90-item symptom list (scl90 test report), Hamilton Anxiety Scale

Table S4: International database and terminology standards for generating

standard data elements

Category	Standards
Patient demographic information	National Health Industry Standard WS445.10-2014 Electronic Medical Record Inpatient Medical Record Homepage
	SEERPCSM2015
	FORDS2015
	NCDRRV52
Medical records	National Health Industry Standard WS445.10-2014 Electronic Medical Record Inpatient Medical Record Homepage
	SEERPCSM2015
	FORDS2015
	NCDRRV52
One complaint and five histories	National Health Industry Standard WS445.12-2014 Electronic Medical Records Inpatient Admission Records
Physical examination	Medical Record Writing Specifications 2010 Edition
Specialist examination	HL7 China CDA Discharge Summary Trial Implementation
Diagnosis	EER PCSM 2015, FORDS 2015
	Diagnosis names follow ICD-10 V6.01, MESH, ICD-O-3 standards for normalization and standardization.
Test	People's Republic of China Health Industry Standard WS445.4-2014 Electronic Medical Record Inspection Record
	The inspection method and item name follow the Loinc standard for normalization and standardization
Examination	People's Republic of China Health Industry Standard WS445.4-2014 Electronic Medical Record Inspection Record
	Inspection methods and project names follow the ICD-9-CM-3 standard for normalization and standardization
Pathology	People's Republic of China Health Industry Standard WS445.4-2014 Electronic Medical Record Inspection Record
Operation	The name of the surgery follows the name of the surgery (operation) in ICD-9-CM-3
Drug	Standardized according to CFDA+FDA+ATC classification
Cardiovascular disease related field set	ESC European Society of Cardiology Guidelines
	ACC American College of Cardiology Guidelines
	Chinese Society of Cardiology Guidelines
	HFA Heart Failure Association Guidelines
	Cardiovascular CRF Form
Other related terminology standards	Systematized Nomenclature of Medicine-Clinical Terms， SNOMED-CT
